# Synergistic activation of grapevine defense mechanisms against downy mildew by *Ascophyllum nodosum* extract and *Pseudomonas fluorescens* CHA0

**DOI:** 10.3389/fpls.2025.1568426

**Published:** 2025-06-05

**Authors:** Ramin Bahmani, Pramod Rathor, Prashant More, Balakrishnan Prithiviraj

**Affiliations:** Marine Bio-Products Research Laboratory, Department of Plant, Food and Environmental Sciences, Faculty of Agriculture, Dalhousie University, Truro, NS, Canada

**Keywords:** biostimulant, defense enzymes, induced systemic resistance, jasmonic acid signaling, pathogen-responsive genes

## Abstract

Downy mildew, caused by *Plasmopara viticola*, poses a major threat to grapevine production, necessitating effective and sustainable disease management strategies. *Ascophyllum nodosum* extract (ANE) and *Pseudomonas fluorescens* CHA0 possess biostimulant and biocontrol properties, respectively, with reported potential to induce plant defense against various pathogens. However, their combined application and potential synergistic effects in grapevine disease management remain largely unexplored. In this study, their efficacy in controlling downy mildew in grapevines was investigated. Our results demonstrate that both ANE and CHA0, individually and in combination, significantly reduced downy mildew severity. *In vitro* assays revealed that grape leaves treated with ANE, CHA0, or their combination suppressed disease establishment and progression, with the combined treatment showing the greatest reduction in spore count (~66%), zoospore numbers (~89–93%), and empty sporangia (~53–69%). *In vivo* greenhouse experiments confirmed these findings, showing that combined foliar applications of ANE and CHA0 reduced downy mildew incidence to approximately 22%, compared to 40–50% with individual treatments and 70% in untreated controls, implying a synergistic interaction between these treatments. In addition to disease suppression, combined ANE and CHA0 applications triggered robust biochemical and molecular defense responses in grapevines. Key defense enzymes, including phenylalanine ammonia lyase, peroxidase, and polyphenol oxidase, showed significantly enhanced activities, accompanied by elevated levels of phenolics and hydrogen peroxide. At the molecular level, the combination significantly upregulated stress-responsive genes such as *CHI*, *GLP2* and *GLP7*, along with jasmonic acid-responsive genes including *LOX9*, *OSM1*, and *PR4*, suggesting a priming effect that reinforces the plant’s innate immune responses. Overall, these findings highlight the potential of combined ANE and CHA0 treatments in enhancing grapevine resistance to downy mildew through the activation of defense mechanisms and modulation of key biochemical markers. The synergistic interplay between the biostimulant and biocontrol agent offers a promising eco-friendly alternative to conventional chemical-based disease management in viticulture.

## 1 Introduction

Grapes (*Vitis vinifera* L.) are one of the most widely cultivated fruit crops worldwide. They can be consumed fresh, dried to make raisins, crushed for juice, or fermented into alcoholic beverages, highlighting the grape industry’s extensive reach and economic significance. With increasing popularity, grapes have become the world’s third most valuable horticultural crop (Alston and Sambucci, 2019). Over the past decade, the global grape industry has experienced rapid growth, with grape production increasing from 69.1 million to 73.5 million tons (FAO, 2022). However, grape productivity is affected by various biotic and abiotic stresses, with downy mildew caused by the oomycete *Plasmopara viticola* being a significant concern. This pathogen poses a major threat to viticulture, impacting nearly all grapevine varieties. Symptoms of the disease include yellow circular spots on the upper surface of leaves and white, downy fungal growth on the lower surface (Peng et al., 2023). Although fungicides are crucial for managing downy mildew, their overuse has contributed to the resurgence of the pathogen and raised environmental concerns. Additionally, the growing demand for organic grapes and grape-based products is driving growth in the global organic grape market (Jindo et al., 2022). Therefore, exploring environmentally friendly alternatives for disease management is essential to minimize the chemical impact on grape production and promote sustainable practices.

An effective alternative to the use of pesticides and synthetic fertilizers is the application of biostimulants and biologicals. Biostimulants, widely used in organic vineyards, can reduce dependence on synthetic fertilizers, pesticides, and fungicides. Among these, seaweed extracts are particularly notable for their environmental sustainability, efficacy, and long shelf life. *Ascophyllum nodosum* extracts (ANE) have been extensively researched for their beneficial effects on plant health. At low concentrations, these extracts enhance plant resistance to a wide range of fungal and bacterial pathogens (Jardin, 2015; Bajpai et al., 2019; Bahmani et al., 2023b). *A. nodosum* contains several biologically active components, including phlorotannins, alginic acid, fucoidans, and laminarin (Shukla et al., 2019). Previous studies have demonstrated that seaweed extracts from various species can effectively control downy mildew in grapes. Raj et al. (2019) investigated the effects of extracts from brown, green, and red seaweeds on downy mildew and found that the extract from *Hydroclathrus clathratus*, a brown seaweed, was the most effective in preventing the disease. Extracts from *A. nodosum* have been shown to enhance grape growth by improving copper uptake (Turan and Köse, 2004) and increasing freezing tolerance (Wilson, 2001). However, to date, there have been no documented reports of using *Ascophyllum* extracts for managing downy mildew in grapevines.

In addition to biostimulants, biocontrol agents have also proven effective in managing different plant diseases. Different species of *Pseudomonas* have biocontrol properties and have been found to control various plant pathogens. *Pseudomonas fluorescens* is a beneficial plant growth-promoting rhizobacterium (PGPR) known for enhancing plant health through induced systemic resistance and biological control of pathogens (Ganeshan and Kumar, 2005). Different strains of *P. fluorescens* have been reported to be effective against downy mildew disease in various crops such as maize (Kamalakannan and Shanmugam, 2009), pearl millet (Lavanya et al., 2022), and grapes (Lakkis et al., 2019). The downy mildew of grapevine was controlled by applying *P. fluorescens* PTA-CT2, which induced systemic resistance in the grapevine against *P. viticola* (Lakkis et al., 2019). Currently, there are no reports of *P. fluorescens* CHA0 (hereafter referred to as CHA0) being used to manage *P. viticola* for the control of downy mildew disease. However, CHA0 has demonstrated potentially control several plant diseases caused by soil-borne pathogens (Keel et al., 1992; Jousset et al., 2006).

Plants possess inducible defense mechanisms to cope with pathogen infection challenges (Conrath et al., 2002; Wiesel et al., 2014) including systemic acquired resistance (SAR) and induced systemic resistance (ISR). SAR is mediated by salicylic acid (SA) and the expression of pathogenesis-related (PR) genes, while jasmonic acid (JA) and ethylene (ET) signaling pathways are involved in ISR (Lemarié et al., 2015). Biostimulants and biocontrol agents have been shown to improve the plant’s resistance to pathogens by triggering these mechanisms (Compant et al., 2005; Shukla et al., 2019).

Various defense enzymes play crucial roles in these mechanisms. Among them, polyphenol oxidase (PPO) is involved in lignification and the oxidative stress response, contributing to pathogen resistance by catalyzing the oxidation of phenolic compounds (Hiraga et al., 2001; Mayer, 2006). Peroxidase (PO) reinforces cell walls through the synthesis of lignin and suberin while also generating reactive oxygen species (ROS) to combat pathogen invasion (Almagro et al., 2009). Phenylalanine ammonia-lyase (PAL) is a key enzyme in the phenylpropanoid pathway, facilitating the biosynthesis of secondary metabolites such as phytoalexins and lignin, which enhance structural defense (Potin et al., 1999; Hemm et al., 2004; Kim and Hwang, 2014). In addition to enzymatic defense, pathogenesis-related (PR) proteins contribute to plant immunity through various mechanisms. Lipoxygenase (*LOX*) is involved in the oxidation of polyunsaturated fatty acids and plays a central role in the jasmonic acid (JA) signaling pathway, mediating defense responses against pathogen invasion (Park et al., 2010; Podolyan et al., 2010; Wasternack and Song, 2017). Plant defensins (*PDF*s) exhibit antimicrobial activity by disrupting pathogen membranes, thereby limiting fungal and bacterial infections (Sher Khan et al., 2019). Germin-like proteins (*GLP*s) are a diverse group of water-soluble glycoproteins that provide antifungal activity, participate in oxidative stress responses, and reinforce cell walls, thereby impeding pathogen penetration (Dunwell et al., 2008). Furthermore, thaumatin-like proteins (*TLP*s) act as antifungal agents by disrupting fungal cell membranes and inhibiting pathogen growth (Liu et al., 2010). These proteins function within the salicylic acid (SA)-mediated defense pathway, the jasmonic acid (JA)/ethylene (ET)-mediated defense pathway, or both, depending on the nature of the pathogen and the plant species (Thomma et al., 1998; Zhu et al., 2002; Lemarié et al., 2015; Yan et al., 2017). Previous studies have shown that enhanced plant resistance to pathogens is often correlated with elevated activities of these defense enzymes and increased expression of pathogenesis-related (PR) genes (Jayaraj et al., 2008; Subramanian et al., 2011; Esserti et al., 2016; Bahmani et al., 2023b).Despite reports highlighting the effectiveness of ANE and CHA0 in controlling various plant diseases, their potential for managing downy mildew in grapevine remains unexplored. Additionally, potential synergistic interactions between ANE and CHA0 have not been investigated.

The goals of the current study were to investigate the antifungal potential of ANE and CHA0 through both direct and indirect modes of action. This included conducting *in vitro* assays using the leaf disc treatment method and spore assays, studying the defense mechanisms in grapevines treated with ANE and CHA0, either alone or in combination, and evaluating the effectiveness of their combined application in reducing downy mildew symptoms. The results demonstrated that the application of ANE and CHA0 inhibited the occurrence and spread of *Plasmopara viticola* and enhanced grape resistance to downy mildew by activating various plant defense mechanisms.

## 2 Materials and methods

### 2.1 Plant material and growth condition

Grapevines of the Chardonnay variety were obtained from VineTech, Canada. The grapevines were acclimated and potted in Pro-Mix. The vines were grown in a greenhouse (Conviron, Canada) maintained at 70–80% relative humidity (RH) and a day/night temperature of 24/21 ± 2°C with a 16-h light/8-h dark photoperiod. Plants were irrigated with a fertilizer solution (1 g/L of 20-20–20 nitrogen, phosphorus, and potassium (NPK)) at 50 mL per plant once every two weeks. Pots were irrigated with water on alternate days or as needed.

### 2.2 Isolation and maintenance of disease inoculum

Inoculum of downy mildew (*Plasmopara viticola*) was collected from naturally infected grapevines in vineyards located in Wolfville, Nova Scotia, Canada. The inoculum was maintained by repeated inoculations on Chardonnay grape plants grown under greenhouse conditions, as described above. The *P. viticola* spore suspension was prepared in sterile distilled water and sprayed on the abaxial surface of the grape leaves. After spraying, the plants were placed in a mist chamber with continuous misting at 21°C overnight with >90% RH. After 12 hours, the misting was stopped, and the plants were subjected to dry conditions. This cycle was repeated every 12 hours for seven days. After seven days, infection symptoms appeared on the grape leaves as sporulating lesions on the abaxial surface. Sporulation increased until day 10. Leaves with freshly sporulating lesions were collected and washed in cold (4°C) sterile distilled water to collect the sporangia. The sporangia suspension was filtered through sterile cotton wool and adjusted to a concentration of 2 × 1^5^ spores/mL by counting with a hemocytometer.

### 2.3 *Pseudomonas protegens* CHA0 and ANE treatments

The *Pseudomonas protegens* CHA0 (hereafter referred to as CHA0) strain was obtained from Dr. Zhenyu Cheng at Dalhousie University. CHA0 was cultured and maintained on King’s B agar (10 g agar, 20 g peptone mix, 1.5 g anhydrous K_2_HPO_4_, 1.5 g MgSO_4_, and 10 mL glycerol in 1 L distilled water, pH 7.2) at 28 ± 2°C. A glycerol stock solution was prepared from this culture and used to prepare a bacterial suspension of CHA0 at a concentration of 10^7^ CFU/mL. ANE (Acadian Seaplants Limited) stock solution (10%) was prepared in distilled water and stored at 4°C until further use. Foliar treatments were applied until runoff occurred, and soil drench applications were performed by adding 400 mL of either ANE or CHA0 to achieve the desired final concentration.

### 2.4 Leaf disc assay

The disc assay was performed using the moist-chamber Petri dish method with minor modifications (Cohen, 1993). Leaf discs (1 cm in diameter) were placed in Petri dishes lined with two layers of Whatman filter paper wetted with 8 mL of the treatment solution, which included ANE (0.01% v/v), CHA0 (10^7^ CFU/mL), and their combination. For the combination treatment, 800 µL of CHA0 from a 10^8^ CFU/mL stock and 800 µL of ANE from a 0.1% (v/v) stock were combined in 8 mL of King’s B liquid medium to achieve final concentrations of 10^7^ CFU/mL for CHA0 and 0.01% (v/v) for ANE, respectively. Distilled water was used as the control treatment. Each leaf disc was floated on the treatment solution. A 50 µL aliquot of the downy mildew spore suspension was placed at the center of each leaf disc. To maintain humidity, the plates were sealed with Parafilm and incubated in the dark at 21°C for 24 h. After 24 h, the suspension droplet was removed using sterile filter paper to prevent secondary contamination, and the plates were returned to the incubator for 7 days. At the end of the incubation period, leaf discs were examined for sporulation and disease symptoms. Each treatment was performed with three independent biological replicates, with each replicate consisting of five leaf discs.

### 2.5 Spore assay

The downy mildew spore suspension was amended with ANE (0.01% v/v), CHA0 (10^7^ CFU/mL), and their combination, then incubated at 18°C. The suspension was observed under a microscope at 2 h and 6 h intervals. The number of released and actively swimming zoospores was counted at 10× magnification using a hemocytometer. Additionally, the number of empty sporangia was recorded and compared across treatments. Each treatment was performed with three independent biological replicates.

### 2.6 Downy mildew disease evaluation under greenhouse condition

The downy mildew disease evaluation was performed as previously described by Lazazzara et al. (2021) with some modifications. The grape plants were grown and maintained in the greenhouse as described above. Uniform, healthy plants were selected for the experiments. The plants were treated as follows: 0.1% ANE as a foliar spray, 0.3% ANE as a soil drench, CHA0 as a soil drench (10^7^ CFU/g of soil), CHA0 as a foliar spray (10^7^ CFU/mL), 0.1% ANE + CHA0 (10^7^ CFU/mL) as a foliar spray, 0.3% ANE + CHA0 (10^7^ CFU/g of soil) as a soil drench, non-treated plants were used as controls. Six hours after treatment application, plants were inoculated with a *P. viticola* spore suspension (2 × 1^5^ spores/mL) on the abaxial surfaces of the leaves. Following pathogen inoculation, the plants were incubated in a mist chamber under continuous mist in dark conditions at 21°C overnight with >90% RH. After 12 h, the misting was stopped, and plants were transferred to controlled greenhouse conditions as previously described. The severity of the disease was visually assessed based on the percentage of abaxial leaf area covered by sporulation and scored according to the protocol described by European and Mediterranean Plant Protection Organization (2001) as follows: 1 = No infection, 2 = < 5% leaf area infected, 3 = 5–10% leaf area infected, 4 = 10–25% leaf area infected, 5 = 25–50% leaf area infected, 6 = 50–75% leaf area infected, 7 = >75% leaf area infected. Five replicates were analyzed for each treatment, and the experiment was conducted three times independently.

### 2.7 Plant growth and treatments for gene expression analysis and biochemical assays

Three-month-old, uniform, and healthy grape plants were treated with 0.1% ANE, CHA0 (10^7^ CFU/mL), and 0.1% ANE + CHA0 (10^7^ CFU/mL) as a foliar spray and maintained in the greenhouse, as described above. Leaf samples were harvested at 24, 48, and 72 h post-treatment, ground in liquid nitrogen, and stored at −80°C for gene expression analyses and biochemical assays.

### 2.8 Gene expression analysis

The expression of defense-related genes was examined using qRT-PCR. Total RNA was isolated from the leaf samples (100 mg) using TRIzol reagent (Invitrogen) following the manufacturer’s instructions. Two micrograms of RNA were treated with DNase (Promega) and converted to cDNA using a RevertAid cDNA Synthesis Kit, following the manufacturer’s instructions. The qRT-PCR analysis was performed as previously described (Bahmani and Prithiviraj, 2024) using a StepOne Plus Real-Time PCR System (Applied Biosystems). Gene-specific primer sequences were designed using Primer3 Plus Software. ACTIN was used as the endogenous control for normalization. The experiment was performed with three independent biological replicates, each consisting of three technical replicates. The sequences of gene-specific primers are provided in **Supplementary Table S1**.

### 2.9 Assessment of peroxidase and polyphenol oxidase activity

Briefly, ground leaf samples (500 mg) were extracted in 50 mM sodium phosphate buffer supplemented with 0.05% polyvinylpyrrolidone (PVP). The homogenate was centrifuged at 12,000 rpm for 15 minutes at 4°C, and the resulting supernatant was collected as the crude enzyme extract. Total protein content was determined using the Bradford assay (Bradford, 1976). Peroxidase (PO) and Polyphenol Oxidase (PPO) activities were measured using guaiacol and catechol as substrates, respectively, following the previously described method (Ngadze et al., 2012). For PO activity, the reaction mixture contained 0.5 ml of crude enzyme extract, 1.5 ml of 50 mM sodium phosphate buffer, 1 ml of guaiacol, and 1 ml of 2% hydrogen peroxide (H_2_O_2_). The absorbance was measured at 470 nm for 5 minutes, with readings taken at 30-second intervals. For PPO activity, the reaction mixture contained 1 ml of crude enzyme extract, 2.9 ml of 50 mM sodium phosphate buffer, and 1 ml of 100 mM catechol (Sigma). The reaction was monitored by measuring absorbance at 546 nm at 30-second intervals for 5 minutes using a Cytation™ 5 multimode plate reader (BioTek, USA). Enzyme activities were expressed as the change in absorbance per minute per milligram of protein. The experiment was performed with three independent biological replicates.

### 2.10 Phenylalanine ammonia lyases activity assay

PAL activity was assessed using 500 mg of leaf tissue and phenylalanine as the substrate following the method described in detail earlier (Subramanian et al., 2011). Enzyme activity was measured as the change in absorbance at 290 nm, corresponding to the conversion of L-phenylalanine to trans-cinnamic acid, using a Cytation™ 5 multimode plate reader (BioTek, USA). The amount of trans-cinnamic acid produced was calculated using a standard curve of trans-cinnamic acid and expressed as nmol of trans-cinnamic acid per mg of tissue. The experiment was performed with three independent biological replicates.

### 2.11 Total phenolic content

Total phenolic content in leaf samples (1 g) was determined using the Folin-Ciocalteu reagent (Zieslin and Ben-Zaken, 1993). Briefly, plant samples were extracted in 85% ice-cold methanol (v/v) and incubated at room temperature in the dark for 48 hours. The homogenate was then centrifuged, and the resulting supernatant was collected and mixed with 10% Folin-Ciocalteu reagent by vortexing. Subsequently, 0.7 M sodium carbonate (Na_2_CO_3_) was added, and the reaction mixture was further incubated for 2 hours. After incubation, the absorbance of the samples was measured at 725 nm using a Cytation™ 5 plate reader (BioTek, USA), and total phenolic content was calculated using a standard curve of gallic acid (0–10 µg/mL). Results were expressed as µg of gallic acid per g fresh weight (µg GAE/g FW). The experiment was performed with three independent biological replicates.

### 2.12 Measurement of hydrogen peroxide

The hydrogen peroxide (H_2_O_2_) content in leaf tissues (100 mg) was assessed using a spectrophotometric method described earlier (Bahmani et al., 2023a) using a Cytation™ 5 plate reader (BioTek, USA). The assay included 500 µL of plant extract supernatant (prepared in 0.1% trichloroacetic acid), 500 µL of 10 mM potassium phosphate buffer (pH 7.0), and 1 mL of 1 M potassium iodide. A blank sample containing all components except the plant extract was used for calibration. H_2_O_2_ levels were quantified using a standard curve of known H_2_O_2_ concentrations. The experiment was performed with three independent biological replicates.

### 2.13 Statistical analysis

Data were analyzed using one-way or two-way analysis of variance (ANOVA), depending on the number of factors involved in each experiment. For experiments involving a single factor, one-way ANOVA was applied (**Supplementary Table S2**). When both treatment and time factors were considered, two-way ANOVA was employed to evaluate their individual effects and potential interactions (**Supplementary Tables S3** and **S4**). *Post-hoc* comparisons were conducted using Tukey’s test, with statistical significance set at *P* ≤ 0.05. All analyses were performed using Minitab software (version 22).

## 3 Results

### 3.1 ANE and CHA0 applications reduced downy mildew disease severity in grapevines

**Figure 1:**
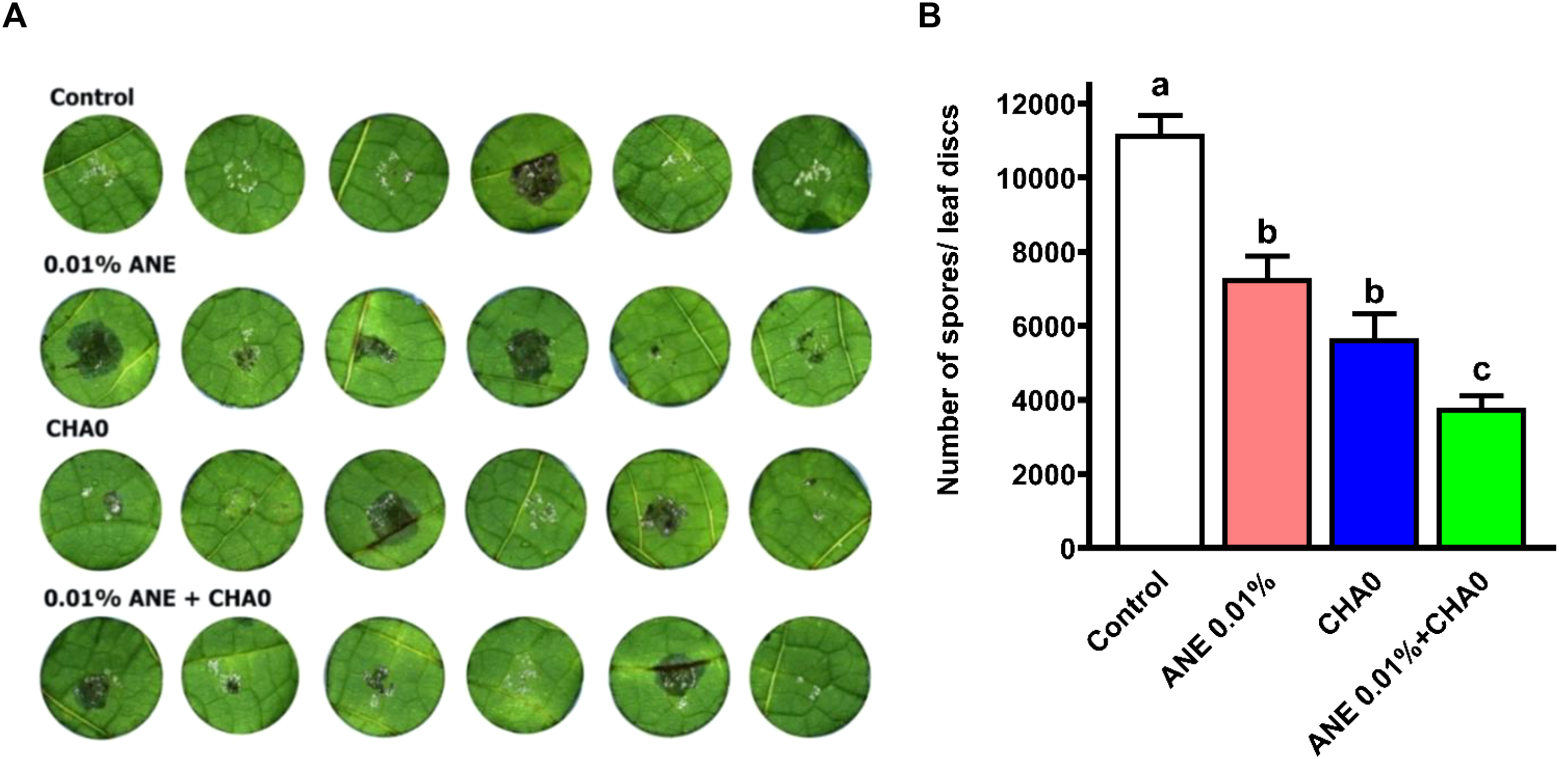
The combined application of *Ascophyllum nodosum* extract (ANE) and *Pseudomonas fluorescens* CHA0 reduces downy mildew disease development on grapevine leaf discs. **(A)** Representative photographs of grapevine leaf discs treated with 0.01% (v/v) ANE, *Pseudomonas fluorescens* CHA0 (10^7^ CFU/mL), and their combination, showing necrotic lesions and downy mildew growth. Leaf discs were inoculated with *Plasmopara viticola* sporangia and incubated for 7 days, after which they were photographed and examined for disease symptoms. **(B)** Bar graph representing the number of *P. viticola* spores per leaf disc following treatment with ANE, CHA0, or their combination. Data were analyzed using one-way ANOVA, and values are presented as the mean ± standard error (SE) from three biological replicates (n = 3). Different letters above the bars indicate significant differences according to Tukey’s test (p ≤ 0.05).

**Figure 2:**
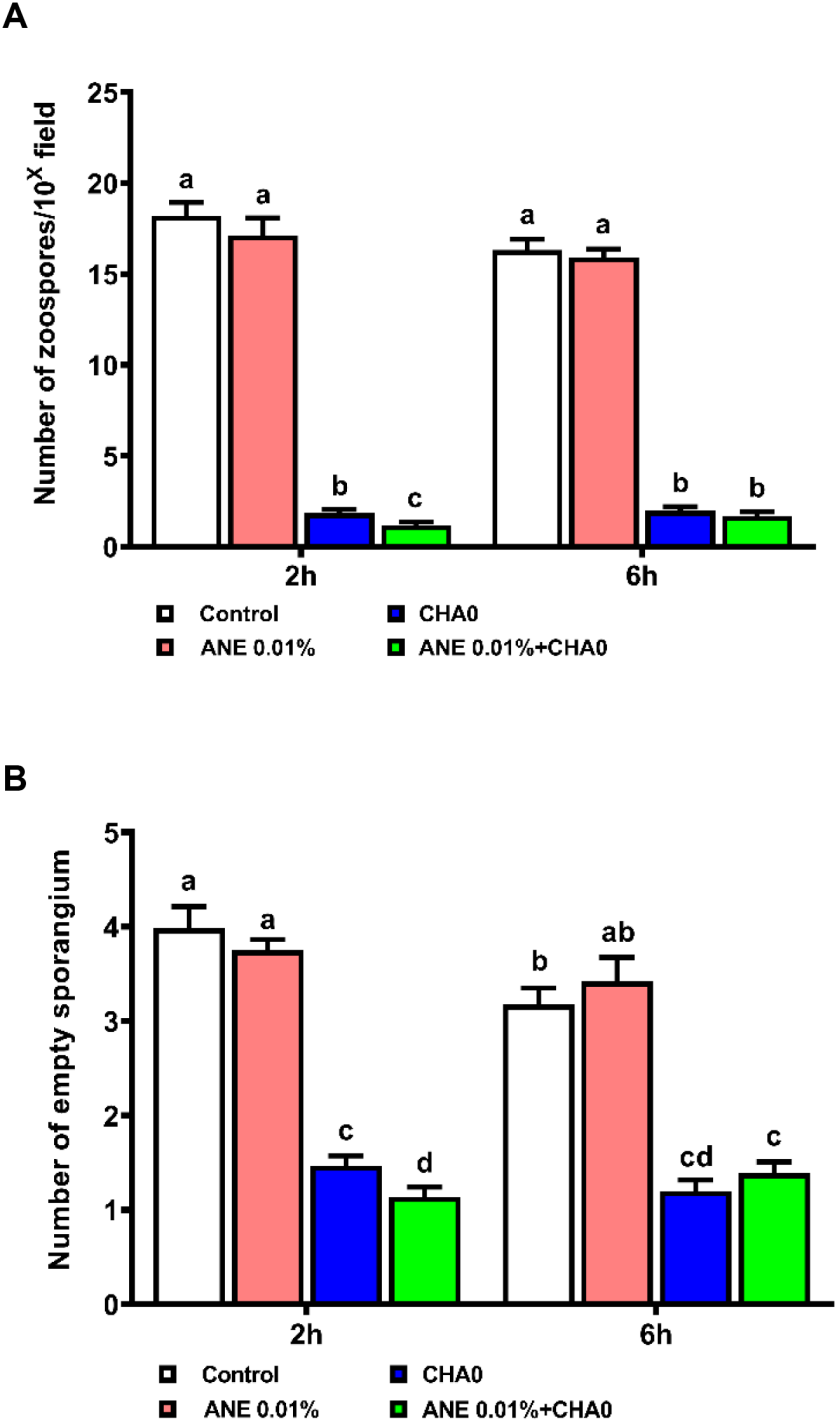
*Ascophyllum nodosum* extract (ANE) and *Pseudomonas fluorescens* CHA0 influence the sporulation dynamics of *Plasmopara viticola* over time post-inoculation. **(A)** Bar graph representing the number of zoospores, and **(B)** the number of empty sporangia, following treatment with 0.01% (v/v) ANE, *Pseudomonas fluorescens* CHA0 (10^7^ CFU/mL), and their combination at 2 h and 6 h post-inoculation. Data were analyzed using two-way ANOVA, and values are presented as the mean ± standard error (SE) from three biological replicates (n = 3). Different letters above the bars indicate significant differences according to Tukey’s test (p ≤ 0.05).

**Figure 3:**
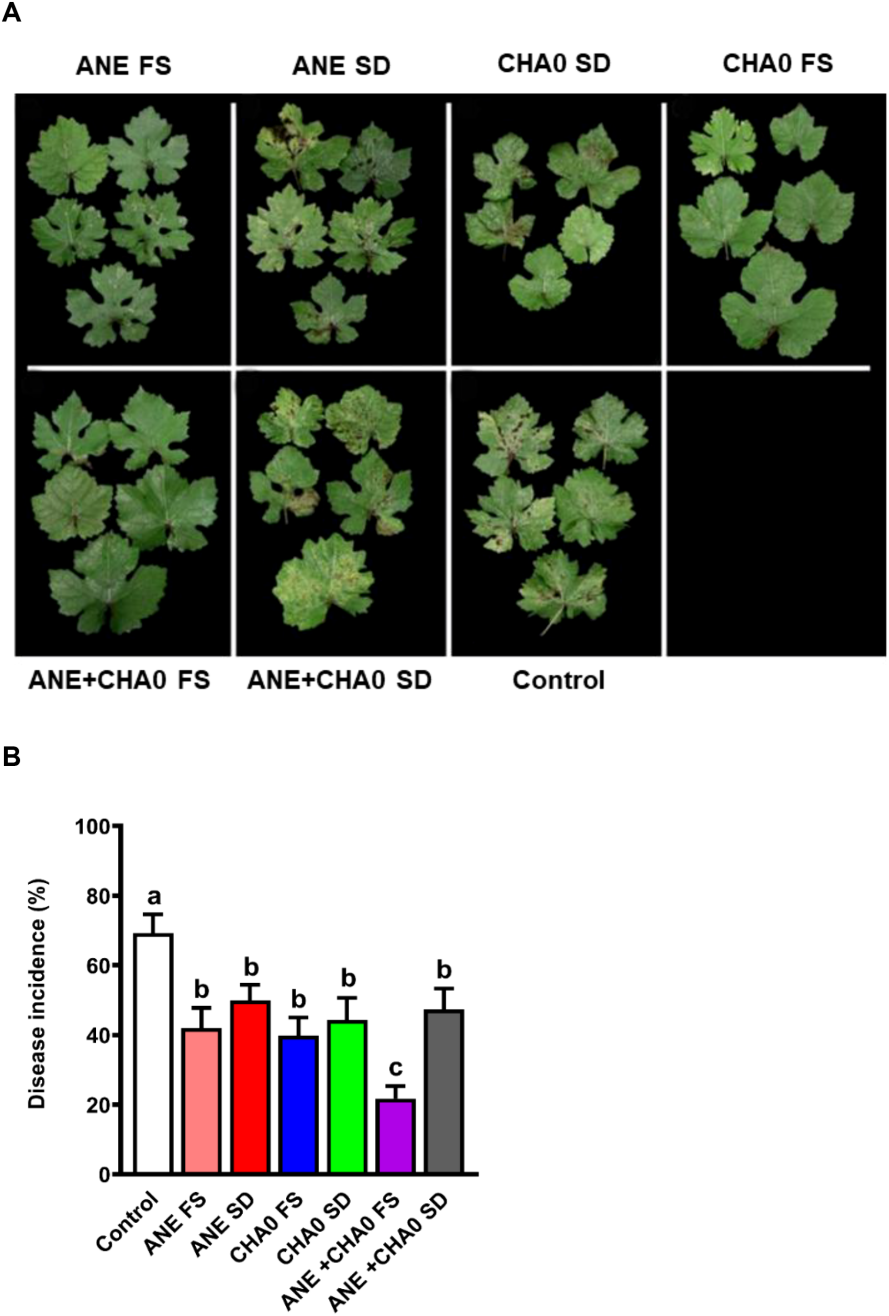
*Ascophyllum nodosum* extract (ANE) and *Pseudomonas fluorescens* CHA0 reduce downy mildew disease incidence in grapevine. **(A)** Representative images of grapevine leaves exhibiting disease symptoms following treatment with ANE, *Pseudomonas fluorescens* CHA0, and their combination, applied via soil drench or foliar spray. The plants were inoculated with a *P. viticola* spore suspension (2 × 1^5^ spores/mL) and maintained under greenhouse conditions as described in Materials and Methods. **(B)** Bar graph indicating disease incidence (%), calculated as the percentage of infected leaves per plant in each treatment. ANE FS: 0.1% ANE foliar spray, ANE SD: 0.3% ANE soil drench, CHA0 SD: CHA0 (10^7^ CFU/mL) soil drench, CHA0 FS: CHA0 foliar spray (10^7^ CFU/mL), ANE+CHA0 FS: 0.1% ANE + CHA0 (10^7^ CFU/mL) foliar spray, ANE+CHA0 SD: 0.3% ANE + CHA0 (10^7^ CFU/mL) soil drench, control: non-treated plants. Data were analyzed using one-way ANOVA, and values are presented as the mean ± standard error (SE) from three biological replicates (n = 5). Different letters above the bars indicate significant differences according to Tukey’s test (p ≤ 0.05).

To examine the effectiveness of ANE and CHA0 in controlling downy mildew in grapevines, pathogen inhibition under *in vitro* conditions and disease incidence under *in vivo* conditions were assessed following their individual and combined treatments. In the leaf disc assay, grape leaves treated with ANE, CHA0, or their combination showed enhanced resistance to downy mildew at 7 days post-inoculation (**Figure 1A**). A significant reduction in spore count was observed in ANE (~35%) and CHA0 (~50%) single treatments compared to untreated control. However, the combined application of ANE and CHA0 resulted in a significantly greater reduction in spore count (~66%) compared to either treatment alone (**Figure 1B**). Further analysis revealed a significant reduction in zoospore numbers and empty sporangia across all treatments at both 2- and 6-hour post-inoculation, except for the 0.01% ANE treatment, where no significant difference was observed compared to the control (**Figures 2A, B**). The combination of 0.01% ANE and CHA0 significantly reduced zoospore numbers and empty sporangia compared to individual treatments at 2 hours post-inoculation. However, at 6 hours post-inoculation, no significant improvement was observed over CHA0 alone (**Figure 2A**). To assess the effectiveness of ANE and CHA0 in controlling downy mildew under greenhouse conditions, disease incidence was evaluated following foliar spray and soil drench treatments, where grape plants were treated with 0.1% and 0.3% concentrations of ANE, CHA0, and their combinations. These treatments exhibited enhanced resistance to downy mildew (**Figure 3A**). Disease incidence was significantly lower across all treatments compared to control plants. No significant differences were observed between the individual treatments of ANE and CHA0. Additionally, soil drench applications of ANE and CHA0 did not differ significantly from their respective foliar applications. However, when ANE and CHA0 were applied in combination, the foliar spray treatment exhibited superior efficacy compared to the soil drench application. Notably, the combined soil drench treatment showed a performance similar to that of the individual ANE or CHA0 treatments, regardless of whether applied as soil drench or foliar spray. Among all treatments, the combined foliar application of 0.1% ANE and CHA0 resulted in the greatest reduction in disease incidence (~22%), which was significantly lower than the control (~70%) and other individual treatments (~40-50%) (**Figure 3B**). Collectively, these results demonstrate the potential of combined ANE and CHA0 treatments in enhancing grapevine resistance to downy mildew.

### 3.2 Enhanced plant defense responses triggered by combined ANE and CHA0 applications

**Figure 4:**
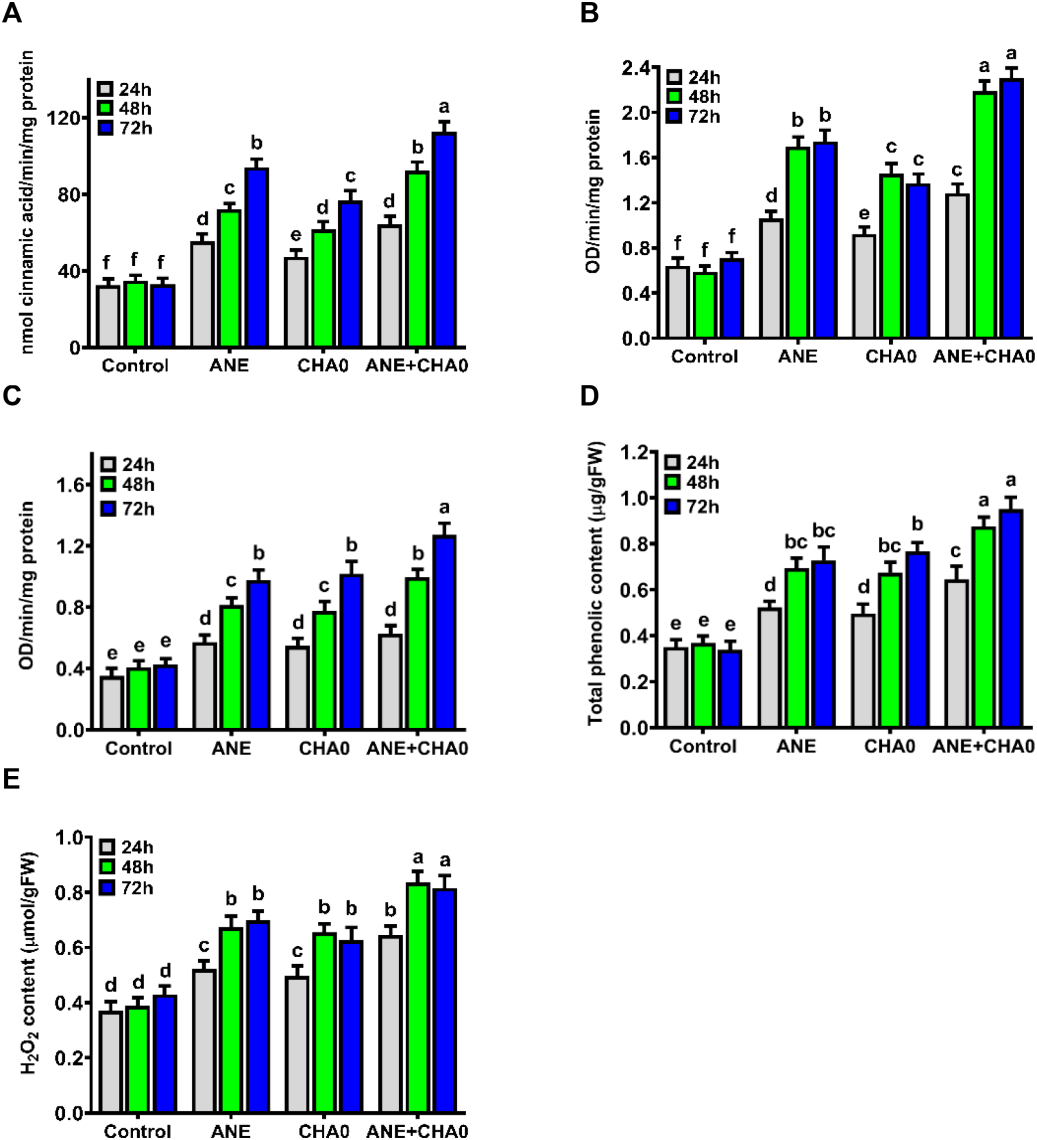
*Ascophyllum nodosum* extract (ANE) and *Pseudomonas fluorescens* CHA0 modulate enzymatic and biochemical defense responses in grapevine. Bar graphs showing the activity of defense-related enzymes and biochemical responses in grapevine leaves following foliar application of 0.1% (v/v) ANE, *Pseudomonas fluorescens* CHA0 (10^7^ CFU/mL), and their combination at 24 h, 48 h, and 72 h post-treatment. **(A)** Phenylalanine ammonia lyase (PAL) activity, **(B)** Peroxidase (PO) activity, **(C)** Polyphenol oxidase (PPO) activity, **(D)** Total phenolic content, and **(E)** Hydrogen peroxide (H_2_O_2_) accumulation. Data were analyzed using two-way ANOVA, and values are presented as the mean ± standard error (SE) from three biological replicates (n = 3). Different letters above the bars indicate significant differences according to Tukey’s test (p ≤ 0.05).

To examine the effect of ANE and CHA0 on plant defense responses, the activity of key defense enzymes and markers in grapevines was assessed over a 72-hour period. Both ANE and CHA0, when applied individually or in combination, synergistically stimulated the activities of phenylalanine ammonia lyase (PAL), peroxidase (PO), polyphenol oxidase (PPO), as well as the total phenolic content (TPC) and hydrogen peroxide (H_2_O_2_) levels. The enzyme activities of PAL, PO, and PPO gradually increased from 24 to 72 hours following treatment, whereas no significant changes were observed in untreated control plants (**Figures 4A, C**). PAL activity was significantly higher in all treatments compared to control plants, with the most pronounced increase observed at 72 hours when ANE and CHA0 were applied in combination. Across all time points, individual treatments of ANE showed higher PAL activity compared to CHA0. The combined treatment resulted in a notable enhancement at 48 and 72 hours, showing a significant increase compared to individual treatments (**Figure 4A**). At 24 hours, no significant differences were observed across the individual or combined treatments (**Figure 4A**). PO activity was significantly increased across all treatments and time points compared to control plants, with the combined application of ANE and CHA0 showing a marked increase at 24, 48, and 72 hours compared to individual treatments (**Figure 4B**). PO activity sharply increased from 24 to 48 hours and then plateaued, with no significant changes thereafter. Similar to PAL activity, individual treatments of ANE showed higher PO activity compared to CHA0 at all time points. PPO activity was significantly elevated in all treatments compared to control plants, with the combined application resulting in the highest levels of PPO activity at 48 and 72 hours compared to individual treatments (**Figure 4C**). At 24 hours, no significant differences were noted between individual and combined applications of ANE and CHA0. Additionally, no significant differences were observed between the individual treatments of ANE and CHA0 at any time point (**Figure 4C**). Like the defense enzyme activities, TPC and H_2_O_2_ content significantly increased from 24 to 48 hours in all treatments, but no significant difference was observed between 48 and 72 hours. These levels remained unchanged in control plants (**Figures 4D, E**). All treatments resulted in a significant increase in TPC and H_2_O_2_ accumulation compared to control plants at all time points (**Figures 4D, E**). While no significant differences were observed between the individual applications of ANE and CHA0, the combined application significantly enhanced TPC and H_2_O_2_ content at all time points compared to their individual treatments (**Figures 4D, E**). These findings demonstrate that treatment with ANE and CHA0 primes the physiological state of grapevines, leading to the effective activation of defense mechanisms and modulation of key biochemical markers associated with plant immunity.

### 3.3 ANE and CHA0 applications altered the expression of genes involved in defense response mechanisms

**Figure 5:**
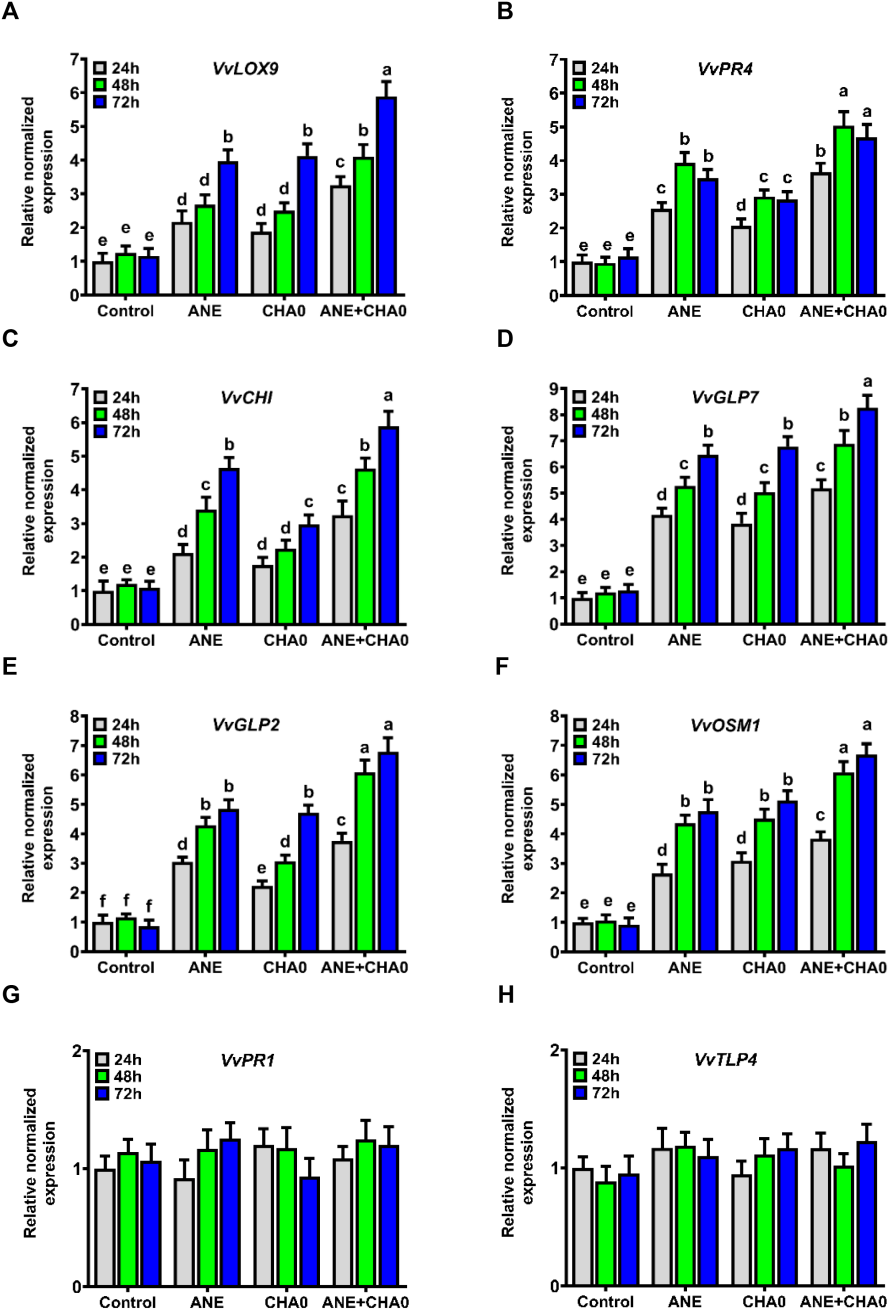
*Ascophyllum nodosum* extract (ANE) and *Pseudomonas fluorescens* CHA0 modulate the expression of pathogen-responsive genes in grapevine. Bar graphs showing the transcript levels of selected pathogen-responsive genes in grapevine leaves following foliar application of 0.1% (v/v) ANE, *Pseudomonas fluorescens* CHA0 (10^7^ CFU/mL), and their combination at 24 h, 48 h, and 72 h post-treatment. Gene expression was analyzed using quantitative real-time PCR (qRT-PCR), with *Actin* used as an endogenous reference gene for normalization. Transcript levels were normalized to the control (water-treated plants). Data were analyzed using two-way ANOVA, and values are presented as the mean ± standard error (SE) of three biological replicates (n = 3). **(A)** *VvLOX9* (lipoxygenase 9), **(B)** *VvPR4* (pathogenesis-related 4), **(C)** *VvCHI* (chalcone isomerase), **(D)** *VvGLP7* (germin-like protein 7), **(E)** *VvGLP2* (germin-like protein 2), **(F)** *VvOSM1* (osmotin 1), **(G)** *VvPR1* (pathogenesis-related 1), **(H)** *VvTLP4* (thaumatin-like protein 4), all genes from *Vitis vinifera* (Vv). Different letters above the bars indicate significant differences according to Tukey’s test (p ≤ 0.05).

Pathogen-responsive genes are essential in plant defense against biotic stresses. To evaluate whether ANE or CHA0 primes grapevine at the molecular level, we quantified the transcript levels of several key pathogen-responsive genes using qRT-PCR. Both ANE and CHA0 treatments, whether individually or in combination, triggered significant molecular responses in grapevines, likely contributing to enhanced defense against downy mildew. The expression of *LOX9*, a JA-responsive gene, was significantly upregulated across all treatments and time points compared to control plants. While no significant differences were observed between the individual treatments of ANE and CHA0 at any time point, *LOX9* expression exhibited a marked increase from 48 to 72 hours in all treatments. Notably, the combined application of ANE and CHA0 resulted in significantly higher *LOX9* expression at all time points compared to their individual treatments, with an early induction observed between 24 and 48 hours (**Figure 5A**). The expression levels of *CHI* and *GLP7* also showed significant upregulation from 24 to 72 hours across all treatments, with no significant changes in untreated control plants (**Figures 5C, D**). For *CHI*, expression was significantly lower in the CHA0 treatment at 48 and 72 hours compared to ANE alone, while no differences were observed at 24 hours (**Figure 5C**). For *GLP7*, no significant differences were observed between the individual treatments of ANE and CHA0. However, the combined treatment consistently showed significantly higher expression levels for both *CHI* and *GLP7* at all time points compared to their individual applications (**Figures 5C, D**).

The expression of *GLP2* and *OSM1* followed a similar pattern, with significantly higher transcript levels in all treatments compared to control plants (**Figures 5E, F**). Both genes showed upregulation from 24 to 48 hours under all treatments, with no further increase at 72 hours, except for *GLP2*, which exhibited significantly higher expression in the CHA0 treatment at 72 hours (**Figure 5E**). Interestingly, *GLP2* expression was significantly lower in the CHA0 treatment at 24 and 48 hours compared to ANE alone, but no differences were observed at 72 hours (**Figure 5E**). For *OSM1*, no significant differences were noted between ANE and CHA0 individual treatments at any time point. However, both *GLP2* and *OSM1* showed significantly higher expression levels under the combined application of ANE and CHA0 compared to the individual treatments at all time points (**Figures 5E, F**). The expression of *PR4* was significantly upregulated across all treatments and time points compared to control plants (**Figure 5B**). This upregulation was most pronounced between 24 and 48 hours, with no significant differences at 72 hours. Additionally, *PR4* expression was consistently lower in the CHA0 treatment compared to ANE alone at all time points. The combined treatment of ANE and CHA0 resulted in significantly higher *PR4* expression compared to the individual treatments (**Figure 5B**). In contrast, the expression of the SA-responsive genes *PR1* and *TLP4* remained unchanged across all treatments, including both individual and combined applications of ANE and CHA0 (**Figures 5G, H**).

Taken together, these findings demonstrate that ANE and CHA0 treatments primarily activate *JA*-responsive genes such as *LOX9*, *PR4*, *GLP2*, and *OSM1*, while SA-dependent pathways remain unaffected. The significant upregulation of *JA*-responsive genes, particularly under the combined application of ANE and CHA0, suggests that these treatments may prime grapevine defense through the *JA* signaling pathway, thereby enhancing the plant’s ability to resist pathogen attacks.

## 4 Discussion

Downy mildew, caused by *Plasmopara viticola*, poses a significant threat to grapevine cultivation worldwide, leading to considerable economic losses. The most common method for controlling this pathogen primarily involves the use of fungicides, which, despite their efficacy, have been associated with adverse effects on soil health, environmental contamination, and the emergence of resistant pathogen strains (Pimentel et al., 1992; Chen et al., 2007). As a result, there is an urgent need for sustainable and less invasive strategies to manage downy mildew in grapevines. This study highlights the effectiveness of ANE and CHA0 in controlling downy mildew in grapevines. Both *in vitro* and *in vivo* trials demonstrated a significant reduction in pathogen establishment and disease incidence following the application of these treatments (**Figures 1**-**3**). Notably, the combined application of ANE and CHA0 consistently outperformed individual treatments, suggesting a synergistic interaction that enhances grapevine resistance to the pathogen. In the greenhouse experiment, applying ANE or CHA0 individually as a foliar treatment was not significantly different from their use as a soil drench. However, when ANE and CHA0 were applied together via foliar application, a substantial improvement was observed compared to the soil drench approach (**Figure 3**). These findings suggest that foliar application is more effective than root drenching, possibly because the elicitor molecules are more mobile or readily available when applied via spraying (Abkhoo and Sabbagh, 2015). The ANE-induced resistance to downy mildew observed in this study can be attributed to the presence of bioactive compounds such as laminarin, fucoidan, alginates, and phytohormones in ANE, which are known to act as elicitors of plant defense pathways (Jayaraj et al., 2008; Sharma et al., 2013; Shukla et al., 2019). By activating signaling pathways involving salicylic acid, jasmonic acid, and ethylene, these compounds trigger ISR or SAR (Subramanian et al., 2011; Ali et al., 2016). The resulting resistance mechanisms enhance the activity of defense enzymes and stimulate the accumulation of phenolic compounds, further strengthening the plant’s ability to resist pathogen invasion (Modafar et al., 2012). Supporting our findings, ANE has been shown to enhance fungal disease resistance in various plants. The application of ANE significantly reduced the incidence of damping-off in cucumber (Abkhoo and Sabbagh, 2015), early blight and bacterial spot in sweet pepper and tomato (Ali et al., 2019), leaf spot, gummy stem blight, and grey mold in cucumber (Jayaraman et al., 2010), powdery mildew in strawberry (Bajpai et al., 2019), and black rot in carrot (Jayaraj et al., 2008).

*Pseudomonas* spp., such as the *Pseudomonas fluorescens* CHA0 strain, are widely recognized as biocontrol agents and have been shown to enhance plant resistance to pathogens by inducing ISR and producing antimicrobial compounds and secondary metabolites (Haas et al., 1991; Haas and Keel, 2003; de Werra et al., 2009; Alattas et al., 2024). Our study demonstrated that the application of *Pseudomonas fluorescens* CHA0 significantly reduced the severity of downy mildew disease in grapevines. This finding is consistent with previous research highlighting the efficacy of CHA0 in controlling various fungal diseases across different plant species, including take-all disease in wheat caused by *Gaeumannomyces graminis* var. *tritici* (Keel et al., 1992; Sari et al., 2008), black root rot in tobacco caused by *Thielaviopsis basicola* (Keel et al., 1992), and damping-off and root rot in cucumber caused by *Pythium ultimum* (Péchy-Tarr et al., 2005).

In the present study, the application of ANE, CHA0, or their combination significantly boosted the activity of key defense enzymes (PAL, PPO, and PO) and the accumulation of phenolic compounds in grapevines. The combined treatment demonstrated a synergistic effect, leading to the most substantial enhancement of these defense traits. These improvements were observed as early as 24 hours post-treatment and remained consistent up to 72 hours (**Figure 4**). The observed activation of defense enzymes can be attributed to the elicitors present in the seaweed extracts, which trigger plant defense mechanisms (Shukla et al., 2019; Bahmani et al., 2023b). The roles of these enzymes in plant defense further support these findings. PAL, a critical enzyme in the phenylpropanoid pathway, facilitates the synthesis of phenolic compounds, phytoalexins, and lignin, which are essential for reinforcing cell walls and hindering pathogen progression (Potin et al., 1999; Hemm et al., 2004; Kim and Hwang, 2014). Similarly, the observed increase in PO activity contributes to lignification and cell wall cross-linking, strengthening plant tissues against enzymatic degradation by pathogens (Bradley et al., 1992). Enhanced PPO activity, which catalyzes the oxidation of phenolic compounds into antimicrobial quinones, underscores the activation of plant defenses induced by these treatments (Hiraga et al., 2001; Mayer, 2006). These enzymatic responses are consistent with prior studies on ANE-treated plants, where increased activities of PAL, PPO, and PO were linked to enhanced resistance against fungal diseases, including early blight (*Alternaria solani*) in sweet pepper and tomato (Ali et al., 2016), powdery mildew (*Podosphaera aphanis*) in strawberry (Bajpai et al., 2019), Alternaria black rot (*Alternaria radicina*) in carrot (Jayaraj et al., 2008), and fusarium root and stem rot (*Fusarium oxysporum*) and botrytis blight (*Botrytis cinerea*) in cucumber (Jayaraman et al., 2010). Furthermore, CHA0-induced enhancement of these enzyme activities highlights its role in ISR, a mechanism by which beneficial microbes prime the plant’s immune system (Bakker et al., 2007). Similar findings have been reported in other studies, where P. fluorescens-mediated resistance to take-all disease in wheat (Sari et al., 2008) and foot rot disease in black pepper (Paul and Sarma, 2005) was correlated with increased activities of defense enzymes such as PAL, PPO, and PO.

In addition to enzymatic defense, the increased phenolic content observed in this study (**Figure 4**) highlights the activation of secondary metabolism by ANE and CHA0, which likely contributed to mitigating downy mildew disease. This increase in phenolic content can be attributed to the activation of phenylpropanoid pathway enzymes such as PAL, PPO, and PO, whose induction was also evident in this study. These enzymes play pivotal roles in phenolic metabolism, with their elevated activity likely contributing to an expanded free phenolic pool. Beyond their antimicrobial activity, phenolic compounds also promote lignin biosynthesis, reinforcing the cell wall and establishing stronger physical barriers against pathogen penetration. The dual functionality of phenolic compounds, induced by ANE and CHA0, enhances the plant’s overall defense response and resistance to downy mildew (Vidhyasekaran, 2007; Jayaraman et al., 2010; Miedes et al., 2014; Carvalho et al., 2018). Consistent with our findings, previous studies have demonstrated that enhanced phenolic content in plants treated with ANE (Jayaraman et al., 2010; Ali et al., 2019; Bajpai et al., 2019) and *Pseudomonas fluorescens* (Paul and Sarma, 2005; Sari et al., 2008) is associated with improved resistance to various fungal pathogens.

The increase in H_2_O_2_ content observed in grapevines treated with ANE and CHA0 (**Figure 4**) likely results from the activation of plant innate immunity mechanisms. Both ANE and CHA0 appear to trigger the production of reactive oxygen species (ROS) as part of the plant’s early defense response to pathogen attack. Among ROS, H_2_O_2_ plays a central role in signaling the plant’s immune system and enhancing resistance to pathogens. It contributes to defense by participating in the oxidation of membrane lipids, which initiates the synthesis of various antifungal substances, such as phytoalexins, reinforcing cell walls, and directly inhibiting pathogen growth by damaging cellular structures (Foyer and Noctor, 2005; Dumanovic et al., 2020). Our findings align with earlier studies demonstrating that treatment with ANE and other brown seaweed extracts induces H_2_O_2_ production, thereby enhancing resistance to fungal and bacterial pathogens (Jayaraj et al., 2008).

Furthermore, single or combined treatments with ANE and CHA0 notably upregulated several pathogen-responsive genes, including *LOX9*, *CHI*, *GLP2*, *GLP7*, *PR4*, and *OSM1*, whereas the expression of *PR1* and *TLP4* did not change significantly (**Figure 5**). This induction was particularly pronounced when ANE and CHA0 were applied together (**Figure 5**). The enhanced expression of these genes in response to ANE and CHA0 treatment correlates with the observed resistance of grapevine in this study. Notably, previous studies have shown that downy mildew-resistant grapevines exhibit higher expression levels of these pathogen-responsive genes (Gessler et al., 2011; Malacarne et al., 2011), further supporting the role of ANE and CHA0 in activating defense pathways. *PR1* and *TLP4* (thaumatin-like proteins) are pathogenesis-related (PR) proteins involved in systemic acquired resistance (SAR), mediating pathogen resistance through their antifungal activities. These proteins hydrolyze the cell walls of invading fungi and disrupt fungal cell membrane integrity, thereby inhibiting fungal growth (Leubner-Metzger and Meins, 1999; Liu et al., 2010; Ali et al., 2018; Goyal et al., 2021). These genes are widely used as markers of salicylic acid (SA) signal transduction pathways, with their upregulation attributed to the accumulation of SA and the activation of SA-mediated defense responses (van Loon et al., 2006; Dufour et al., 2012). Interestingly, neither ANE nor CHA0 induced the expression of these genes, suggesting that ANE/CHA0-mediated enhanced resistance of grapevine to downy mildew is independent of the SA pathway and may not act through SAR (**Figure 5**). This could be explained by the presence of polysaccharides such as laminarin in ANE, which are known to impair SA accumulation and, consequently, affect the expression of these genes (Mercier et al., 2001). These findings are consistent with previous studies showing that disease suppression by ANE treatment does not rely on the SA-dependent pathway (Subramanian et al., 2011; Ali et al., 2016).

The upregulation of jasmonic acid (JA)-responsive genes, including *LOX9*, *PR4*, and *OSM1*, by ANE and CHA0 demonstrates the activation of the JA signaling pathway, which plays a crucial role in plant defense mechanisms (Xu et al., 1994; Thomma et al., 1998; Dufour et al., 2012; Ali et al., 2018). *LOX9* is a lipoxygenase enzyme involved in the oxidation of polyunsaturated fatty acids, contributing to the synthesis of bioactive molecules such as jasmonic acid and other oxylipins, which are essential for the plant’s defense against pathogen invasion (Park et al., 2010; Podolyan et al., 2010; Wasternack and Song, 2017). Similarly, *PR4*, a pathogenesis-related protein with chitinase activity, directly supports plant defense by breaking down the cell walls of invading fungal pathogens, thereby inhibiting their growth (Enoki and Suzuki, 2016). Furthermore, *OSM1*, which encodes osmotin, a PR-5 family protein, plays a crucial role in plant defense under stress by activating the MAPK pathway, disrupting cellular structures, inducing ROS-mediated cell death, and exhibiting antifungal activity (Freitas et al., 2015; Hakim et al., 2018). In agreement with our findings, the expression of *LOX* isoforms in Arabidopsis, *VpPR4* in grapevines, and *OsOSM1* in rice has been shown to enhance resistance to Alternaria leaf spot, powdery mildew, and sheath blight, respectively (Hwang and Hwang, 2010; Dai et al., 2016; Xue et al., 2016). Chalcone isomerase (*CHI*) is an essential enzyme in the flavonoid biosynthesis pathway, significantly contributing to plant defenses against a range of pathogens (Liu et al., 2015). *CHI*-overexpressing soybean plants demonstrated higher resistance to the *Phytophthora sojae* pathogen (Zhou et al., 2018). *CHI* expression can be enhanced by both the SA and JA pathways (Yin et al., 2019). However, since SA and JA often exhibit antagonistic interactions (Yang et al., 2019) and given that the SA-responsive gene examined in this study showed no significant change, the increased expression of *CHI* observed is more likely associated with the activation of the JA signaling pathway. Germin-like proteins (*GLPs*) represent a broad group of water-soluble glycoproteins found across a wide range of plant species (Barman and Banerjee, 2015). They play a crucial role in plant defense by reinforcing cell walls, exhibiting antifungal properties, and mediating oxidative stress responses. Through the production of reactive oxygen species (ROS), regulation of physiological processes, and their enzymatic activity, *GLP2* and *GLP7* promote plant defense responses against pathogens (Bernier and Berna, 2001; Dunwell et al., 2008). Therefore, the priming effect of ANE and CHA0 in increasing the expression of *GLP2* and *GLP7* can be linked to the increased resistance of grapevine to downy mildew observed in the present study. Supporting this, the overexpression of *OsGLP2–1* in rice enhanced resistance to leaf blast, panicle blast, and bacterial blight by stimulating SOD enzyme activity and increasing H_2_O_2_ production (Liu et al., 2016). Moreover, the overexpression of *OsRGLP1* in Medicago truncatula, and *GhGLP2* in cotton has been shown to enhance resistance to *Fusarium oxysporum* and *Verticillium* wilt, respectively (Sultana et al., 2016; Pei et al., 2020). In addition, the enhanced H_2_O_2_ content observed in this study can be correlated with higher expression of *GLP* genes, as the involvement of many *GLPs* in H_2_O_2_ production has been reported (Barman and Banerjee, 2015).

In support of our data, increased transcript levels of pathogen-responsive genes including *CHI*, *PR1*, and *PR5*, have been associated with ANE-induced resistance in carrot plants against the fungal pathogens *Alternaria radicina* and *Botrytis cinerea* (Jayaraj et al., 2008). Similarly, treatment with Stimplex™, a seaweed extract derived from *Ascophyllum nodosum*, reduced the incidence of fungal diseases such as *Alternaria cucumerinum*, *Didymella applanata*, *Fusarium oxysporum*, and *Botrytis cinerea* in cucumber, and this resistance was linked to the upregulation of *CHI*, and *LOX* defense genes (Jayaraman et al., 2010). The activation of these genes by CHA0 treatment is consistent with findings from other studies, where *Pseudomonas fluorescens* PTA-CT2 treatment induced stress-responsive genes, including *LOX9*, *CHI*, and *PR1/6*, in the presence or absence of *Botrytis cinerea* and *Plasmopara viticola* pathogens in grapevines (Gruau et al., 2015; Lakkis et al., 2019). Similarly, tomato plants inoculated with *Pseudomonas putida* exhibited increased levels of *LOXD* and *LOXF* genes (Mariutto et al., 2011). Overall, the priming effect of ANE and CHA0 in grapevine against downy mildew observed in this study appears to be primarily dependent on the jasmonic acid (JA) pathway rather than the salicylic acid (SA) pathway. This observation is intriguing given that *Plasmopara viticola*, the causal agent of grapevine downy mildew, is a biotrophic pathogen typically associated with SA-mediated resistance (Glazebrook, 2005; Pieterse et al., 2009; Peng et al., 2023). However, the role of JA signaling in grapevine resistance against *P. viticola* has been documented (Figueiredo et al., 2015), suggesting that JA-mediated pathways can also contribute to defense against this biotrophic pathogen. This aligns with the complex interplay between SA and JA signaling pathways, which are known to exhibit both antagonistic and synergistic interactions (Pieterse et al., 2014).

## 5 Conclusion

In conclusion, the integrated application of ANE and CHA0 significantly enhances grapevine resilience against downy mildew by inducing the defensive enzyme activities and secondary metabolites, and upregulating pathogen-responsive genes. The synergy between ANE and CHA0 suggests a promising pathway for developing highly effective biocontrol strategies that leverage the strengths of natural products and microbial inoculants. The adoption of such strategies can contribute to the development of sustainable and environmentally friendly disease management practices in viticulture and beyond. Future research should extend to field trials to confirm these promising results and further elucidate the molecular mechanisms involved in these enhanced defensive responses in grapevines.

## Data Availability

The raw data supporting the conclusions of this article will be made available by the authors, without undue reservation.
