# Supplementary material for: Synergistic activation of grapevine defense mechanisms against downy mildew by Ascophyllum nodosum extract and Pseudomonas fluorescens CHA0
Source: Front Plant Sci. 2025 Jun 5;16:1568426. doi: 10.3389/fpls.2025.1568426 (PMC12176806; doi:10.3389/fpls.2025.1568426)
Supplement: Supplementary file 1 [file DataSheet1.pdf]

Supplementary Material

Supplementary Table S1. The list of the primer sequences used for qRT-PCR analysis.

Gene name	Primer Sequences
VvACTIN	F: 5′- ATTCCTCACCATCATCAGCA-3′
	R: 5′- GACCCCCTCCTACTAAAACT-3′
VvPR1	F: 5′- ACTTGTGGGTGGGGGAGAA-3′
	R: 5′- TGTTGCATTGAACCCTAGCG -3′
VvPR4	F: 5′- CAGGCAACGGTGAGAATAGT-3′
	R: 5′- ACCACAGTCCACAAACTCGTA-3′
VvTLP4	F: 5′-CTGCAACTTCGATGCGTCAG-3′
	R: 5′-TGCGAATTCGGCTAAGGTGT-3′
VvLOX9	F: 5′- CCCTTCTTGGCATCTCCCTTA-3′
	R: 5′- TGTTGTGTCCAGGGTCCATTC-3′
VvGLP2	F: 5′-CTTGTTTGCTGCCTCACCAC-3′
	R: 5′-TCTTTGGGGCAAGTCTGGAC-3′
VvGLP7	F: 5′-ACCCTCCTCACTTTCACCCT-3′
	R: 5′-AATGAAGCGGTGTTCAGGGT-3′
VvOSM1	F: 5′- CGCTGCGCTAAAGACTACC-3′
	R: 5′- AAAAACCTTGAGTAATCTGTAGCA-3′
VvCHI	F: 5′-CTTTGACGGGTCGCCAGTAT-3′
	R: 5′-AGAATGGAGTTGCCTGGTGG-3′

Supplementary Table S2. One-way ANOVA summary for number of spores and disease incidence.

Source of Variation	df	Number of spores			Source of Variation	df	Disease incidence (DI)		
		MS	F-value	*P*-value			MS	F-value	*P*-value
Treatment (T)	3	29628561	35.05	0.000***	Treatment (T)	6	598.86	7.17	0.0011**
Error	8	845402			Error	14	83.54		

MS: mean square; df: degrees of freedom; ** and *** indicate significance at $P < 0.01$ and $P < 0.001$, respectively.

Supplementary Table S3. Two-way ANOVA summary of enzymatic, biochemical, and pathogen-related traits analyzed in this study.

Source of Variation	df	PAL			PO			PPO			TPC			H_2O_2		
		MS	F-value	*P*-value	MS	F-value	*P*-value	MS	F-value	*P*-value	MS	F-value	*P*-value	MS	F-value	*P*-value
Treatment (T)	3	5091.98	104.85	0.000***	2.58019	144.03	0.000***	0.51975	53.62	0.000***	0.34365	62.51	0.000***	0.21002	53.70	0.000***
Time (Ti)	2	2569.83	52.92	0.000***	1.13133	63.15	0.000***	0.47982	49.50	0.000***	0.12243	22.27	0.000***	0.06909	17.66	0.000***
T×Ti	6	328.47	6.76	0.000***	0.16611	9.27	0.000***	0.04351	4.49	0.003**	0.01576	2.87	0.030*	0.01157	2.959	0.02627*
Error	24	48.56			0.01791			0.00969			0.00549			0.00391		

Source of Variation	df	Number of zoospore			Number of empty sporangia		
		MS	F-value	*P*-value	MS	F-value	*P*-value
Treatment (T)	3	463.746	561.22	0.000***	10.4223	131.82	0.000***
Time (Ti)	1	3.754	4.544	0.0488*	0.5020	6.35	0.023*
T×Ti	3	2.709	3.280	0.0482*	0.2804	3.55	0.039*
Error	16	0.826			0.0791		

MS: mean square; df: degrees of freedom; *, **, and *** indicate significant at $P < 0.05$, $P < 0.01$, and $P < 0.001$, respectively.

Supplementary Table S4. Two-way ANOVA summary of the qRT-PCR analysis conducted in this study.

Source of Variation	df	*LOX9*			*PR4*			*CHI*			*GLP7*		
		MS	F-value	*P*-value	MS	F-value	*P*-value	MS	F-value	*P*-value	MS	F-value	*P*-value
Treatment (T)	3	16.2011	59.33	0.000^{***}	18.4574	87.83	0.000^{***}	20.0031	73.29	0.000^{***}	52.4048	141.41	0.000^{***}
Time (Ti)	2	9.1699	33.58	0.000^{***}	2.6566	12.64	0.000^{***}	7.8315	28.69	0.000^{***}	13.8339	37.33	0.000^{***}
T×Ti	6	0.9917	3.63	0.0104^{*}	0.5849	2.783	0.0337^{*}	1.1187	4.10	0.006^{**}	1.2957	3.50	0.013^{*}
Error	24	0.2731			0.2101			0.2729			0.3706		

Source of Variation	df	*GLP2*			*OSM1*			*PR-1*			*TLP4*		
		MS	F-value	*P*-value	MS	F-value	*P*-value	MS	F-value	*P*-value	MS	F-value	*P*-value
Treatment (T)	3	32.2058	138.90	0.000^{***}	33.1414	127.53	0.000^{***}	0.01852	0.30	$0.825^{n.s}$	0.08037	1.63	$0.209^{n.s}$
Time (Ti)	2	9.8761	42.59	0.000^{***}	9.9803	38.41	0.000^{***}	0.05094	0.83	$0.450^{n.s}$	0.01105	0.22	$0.801^{n.s}$
T×Ti	6	1.6958	7.31	0.000^{***}	1.2963	4.99	0.002^{**}	0.04603	0.75	$0.618^{n.s}$	0.02626	0.53	$0.778^{n.s}$
Error	24	0.2319			0.2599			0.06166			0.04928		

MS: mean square; df: degrees of freedom; n.s, *, **, and *** indicate non-significant, significant at $P < 0.05$, $P < 0.01$, and $P < 0.001$, respectively.
